# Repurposing Ellipticine Hydrochloride to Combat Colistin-Resistant Extraintestinal Pathogenic *E. coli* (ExPEC)

**DOI:** 10.3389/fmicb.2020.00806

**Published:** 2020-05-25

**Authors:** Hao Lu, Manli Liu, Wenjia Lu, Chenchen Wang, Gaoyan Wang, Wenqi Dong, Xiangru Wang, Huanchun Chen, Chen Tan

**Affiliations:** ^1^State Key Laboratory of Agricultural Microbiology, College of Veterinary Medicine, Huazhong Agricultural University, Wuhan, China; ^2^Key Laboratory of Preventive Veterinary Medicine in Hubei Province, The Cooperative Innovation Center for Sustainable Pig Production, Wuhan, China; ^3^Key Laboratory of Development of Veterinary Diagnostic Products, Ministry of Agriculture of the People’s Republic of China, Wuhan, China; ^4^International Research Center for Animal Disease, Ministry of Science and Technology of the People’s Republic of China, Wuhan, China; ^5^Hubei Biopesticide Engineering Research Centre, Hubei Academy of Agricultural Sciences, Wuhan, China

**Keywords:** *mcr-1*, colistin, ellipticine hydrochloride, ExPEC, multidrug-resistant

## Abstract

Extraintestinal pathogenic *Escherichia coli* (ExPEC) strains are the cause of a majority of human extraintestinal infections globally, resulting in enormous direct economic and medical costs. The plasmid-mediated, colistin-resistant gene *mcr-1* has broken through the ultimate defense line against MDR Gram-negative pathogens. There is an urgent need to discover the new compound intended for colistin-resistant *E. coli.* In this study, antibacterial targets of ellipticine hydrochloride (EH) were confirmed by localized surface plasmon resonance (LSPR) and decatenation assay. The LSPR analysis exhibited good binding between EH and *E. coli* topoisomerase IV. In this study, a synergistic effect is obvious in the combination of EH and colistin, to which eight of ten strains showed synergy, while two isolates (20%) showed no difference. The bacteria enumeration analysis of EH treatment group suggested that the decreased bacterial titer can be observed in various tissues of infected mice. EH treatment significantly decreased the levels of a variety of pro-inflammatory factors, such as TNF-α and IL-6. Moreover, other related lesions, such as inflammatory cell infiltration, alveolar interstitial congestion, and edema were observed to be relieved to different extents. This study reveals the anti-*E. coli* potential activities and molecular mechanism of EH and the therapeutical effectiveness of EH application to animals. It provides us with a new option for fighting against multidrug-resistant ExPEC infections in the future.

## Introduction

Extraintestinal pathogenic *Escherichia coli* (ExPEC) is a major pathogenic factor for humans and animals. It can cause a variety of extraintestinal diseases, such as neonatal meningitis, sepsis, and urinary tract disease. Furthermore, the similarity analysis of the ExPEC isolates from infected humans and animals suggested the possibility of cross-infection between different hosts, including birds, companion animals, pigs, and humans, etc. (Moulin-Schouleur et al., 2006). At present, antimicrobial resistance is considered as a serious threat to human health worldwide. Colistin, a polycationic antimicrobial peptide, is a member of the polymyxin family. Meanwhile, colistin is also a kind of effective antibiotics against multidrug-resistant Gram-negative bacteria, and it has been regarded as the last line therapy for severe bacterial infection to date (Rhouma et al., 2016). *Lancet Infectious Diseases* reported the first case of plasmid-mediated colistin resistance mechanism. The resistance gene was subsequently named as *mcr-1* in 2016 (Liu et al., 2016). In this report, the putative structure and action mechanism of *mcr-1* and its emergence in enterobacteriaceae of animal and human isolates are described, and the spread of *mcr-1* from animals to humans is first proposed (Liu et al., 2016). The rapid spread of resistance genes poses great challenges to human life. Currently, there have been no effective drugs against colistin-resistant *E. coli* pathogens. Therefore, it is especially urgent to develop new drugs resistant against colistin-resistant *E. coli*. To shorten the drug development process, the exploitation of already-used drugs was likely to be a feasible method since these existing substances may play a previously unexploited role in a new context (Thangamani et al., 2015).

*Escherichia coli* topoisomerase IV, a bacterial type II enzyme, consists of 2 ParC and 2 ParE subunits, which is a good target for antibacterial chemotherapy for the following reasons (Kato et al., 1990): (1) It is essential in all bacteria for replication and cell division; (2) an accumulation of cleavage complexes has a bactericidal (not just bacteriostatic) effect; (3) Targeting bacterial type II topoisomerases is nonpoisonous or weakly poisonous for human enzymes; additionally, antibacterial agents show a higher level of specificity/selectivity for prokaryotic enzymes than for eukaryotic enzymes by at least three orders of magnitude (Pommier et al., 2010).

Ellipticine, derived from the leaves of apocynaceae plants, is a natural alkaloid. Ellipticine and its analogs were reported to play potent anti-cancer roles by inhibiting DNA topoisomerase II activity (Stiborova et al., 2001). In addition, ellipticine was reported to have suppressed parasitemia in one previous *in vivo* study. The mean survival time (MST) of mice in the treatment group was prolonged (Rocha e Silva et al., 2012). This provides a research basis for the development of ellipticine hydrochloride (EH) as a drug. Therefore, our study aims to measure the antibacterial activity of ellipticine, to reveal its antibacterial action mechanism, and finally, to assess its antibacterial effectiveness and clinical application to infected animal models.

## Materials and Methods

### Antibacterial Activity of Ellipticine Hydrochloride

All strains tested in this study were selected from existing strains in our laboratory. The following strains (Table 1) were used to evaluate the antibacterial activities of EH: *Staphylococcus aureus* (ATCC29213, ATCC43300), *Klebsiella pneumoniae* (CMCC46117), *Listeria monocytogenes* (ATCC19115), *Pseudomonas aeruginosa* (ATCC9027), *Salmonella typhi* (CMCC(B)50071), *E. coli* (ATCC25922), *E. coli* (RS218, a cerebrospinal fluid isolate from a neonate with meningitis), and ten multidrug-resistant extraintestinal pathogenic *E. coli* that were separated from International Research Center for Animal Disease, Ministry of Science, and Technology of the People’s Republic of China. The evidence of these isolates containing *mcr-1* gene were listed in Supplementary Figure S1. The compounds used in this study were purchased from Topscience. The antimicrobial susceptibility test was performed according to the guideline of the Clinical and Laboratory Standard Institute (CLSI, 2012). Broth microdilution was performed in 96-well plates using Miller-Hinton broth (Hopebio, China). Drugs were dissolved in DMSO and then sterilized with a 0.22-μm syringe filter (Millipore, United States). All compounds and control antibiotics (0.0625–128 mg/L) were tested in triplicate.

**TABLE 1:** Strain used in the study.

Strain ID	Phenotypic properties	Source	Ellipticine hydrochloride (mg/L)	Separation from organ
*S. aureus* (ATCC29213)			0.5	
*S. aureus* (ATCC43300)	MRSA		0.5	
*K. pneumonia* (CMCC46117)			4	
*P. aeruginosa* (ATCC9027)			>128	
*E. coli* (ATCC25922)			1	
*L. monocytogenes* (ATCC19115)			4	
*S. typhi* (CMCC(B)50071)			2	
*E. coli* (RS218)			0.5	
*E. coli* (1145)	Resistant to CL, PIP, GM, TET, and C	China (Hu Nan)	1	Lung
*E. coli* (13712)	Resistant to CL, SAM, CTX, GM, TET, and C	China (Hu Bei)	1	Liver
*E. coli* (1209)	Resistant to CL, SAM, CTX, GM, TET, and C	China (Zhe Jiang)	1	Lung
*E. coli* (1341)	Resistant to CL, SAM, CTX, GM, TET, and C	China (Zhe Jiang)	1	Lung
*E. coli* (1704087)	Resistant to CL, SAM, CTX, GM, TET, and C	China (Zhe Jiang)	0.5	Liver
*E. coli* (42)	Resistant to CL, PIP, GM, TET, LEV and C	China (Hu Bei)	2	Lung
*E. coli* (148121)	Resistant to CL, SAM, CTX, GM, TET, and C	China (Hu Bei)	1	Lung
*E. coli* (1411060)	Resistant to CL, SAM, CTX, GM, TET, and C	China (Hu Bei)	1	Lung
*E. coli* (1603043)	Resistant to CL, SAM, CTX, GM, TET, and C	China (Hu Bei)	1	Lung
*E. coli* (1604040)	Resistant to CL, SAM, CTX, GM, TET, and C	China (Hu Bei)	2	Lung

*CL, colistin; AMP, ampicillin; CTX, cefotaxime sodium; GM, gentamicin; TET, tetracycline; C, chloramphenicol; LEV, levofloxacin; SAM, ampicillin sodium; PIP, piperacillin.*

### Time-Kill Assay

We performed a time-kill assay with EH at a concentration of 4 × minimum inhibitory concentration (MIC) in 2-ml round-bottomed tubes to further investigate the bactericidal action against *E. coli*. We first incubated *E. coli* (ATCC25922) until OD(600) = 0.6, and then diluted it with fresh Miller-Hinton broth to a density of 10^6^ cells/ml. We added test compounds at the concentration of 4 × MIC to each tube and incubated mixture solution at 37°C. The control group was added with equal amount of PBS. At the interval of 30 min, we sampled from the tubes, serially diluted samples, and plated them on LA plates. We incubated the plates overnight at 37°C and counted the colonies to measure viability. The assay was performed in triplicate.

### Decatenation Assay

The effects of compounds on the enzyme’s overall catalytic activity were examined by using a decatenation assay. Decatenation assay were performed as described by Nitiss et al. (2012). The 4 μl of 5× topoisomerase IV reaction buffer (Topogen) and 200 ng kinetoplast DNA (kDNA) from Crithidia fasciculate were added into a series of 200-μl microcentrifuge tubes, and subsequently, 40 nM purified topoisomerase IV (Topogen) and different concentrations of ellipticine hydrochloride (8, 16, 32, 64, and 128 mg/L) were added to these tubes. Distilled water was added into the tubes until the final reaction volume in each tube reached 20 μl. Afterward, tube solution was incubated at 37°C for 30 min. The reaction was terminated by adding 2 μl of 10% SDS and 2 μl of 0.5 mg/ml proteinase K solution, followed by incubation at 37°C for 15 min. Finally, 2.4 μl of 10× loading buffer was added into each tube and gel electrophoresis was performed on a 1% agarose gel for 2 h at 5–10 V/cm. All the experiments were repeated three times.

### Localized Surface Plasmon Resonance (LSPR)

The equilibrium-binding constant (K_D_) of EH and *E. coli* topoisomerase IV was determined by Open SPR (Nicoya, Canada) (Yu et al., 2018). Firstly, smooth detection baseline was obtained by flushing the COOH-sensor chip (Nicoya, Canada) covalently immobilized with *E. coli* topoisomerase IV with running buffer (PBS, pH 7.4). After dilution into several different concentrations (1.56, 3.125, 6.25, and 12.5 μM), EH samples (250 μl) were injected into the chips at an ascending order of the concentration. PBS was used as a negative control. A constant flow rate of 20 μl/min was adopted for each cycle. All the obtained data were analyzed with the Trace Drawer software (Ridgeview Instruments AB, Sweden) to achieve kinetic parameters of the binding reactions.

### Combination Testing

Ten colistin-resistant *E. coli* strains were used to test the antagonistic or synergistic effects between EH and colistin. Five *E. coli* strains (both susceptible and resistant isolates for each antibiotic under investigation) were used to test the antagonistic or synergistic effects between EH with ampicillin, tetracycline, cefotaxime sodium, and levofloxacin. MIC of compound in combination with EH was determined at four different concentration gradients with two-fold dilution method, as previously described (Kohno et al., 2007). The fractional inhibition concentration index (FICI) was calculated by using the following function: FICI = (MIC drug A combination/MIC drug A alone) + (MIC drug B combination/MIC drug B alone), where A and B represented the two antibacterial agents tested. If the calculated FICI was less than 0.5 (including 0.5), a synergy effect between the two agents was determined. FICI value between 0.5 and 4 (including 4) indicated indifference effect between them, and FICI greater than 4 indicated an antagonism effect (Tang et al., 2017). All the experiments were repeated three times.

### Colistin-Induced Membrane Permeability Assay

First, we incubated *E. coli* (42) at 37°C until its OD(600) = 0.6. Then, we diluted strains to the concentration of 10^8^ colony forming units (CFU)/mL, and then we added colistin to cell cultures to reach the concentration of 0.125 mg/L (1/2MIC). Subsequently, we added an equal volume of sterile PBS to cell cultures as the control. We measured colistin-induced outer and inner membrane permeability according to the method previously described (Ma B. et al., 2016). We collected, washed, and resuspended strains in the buffer containing 5 mM HEPES and 5 mM glucose (pH = 7.2) at different time points. Afterward, we measured outer membrane permeability of colistin by the uptake amount of 1-N-phenylnaphthylamine (NPN) (Hancock et al., 1991). We incubated the samples with NPN (8 μL from a 500 μM stock in acetone) at 25°C for 30 min. The incubated samples were then transferred to cuvettes. We measured fluorescence intensity of samples using an F-2700 fluorescence spectrophotometer (Hitachi, Japan) at an excitation wavelength of 350 nm and an emission wavelength of 420 nm. We detected the inner membrane permeability of *E. coli* by the uptake amount of propidium iodide (PI) (Yarlagadda et al., 2014). We added the 10 μM PI to the cells and incubated them at 25°C for 30 min. Then, we used a fluorescence spectrophotometer to measure fluorescence intensity of the dye.

### Liquid Chromatography-Mass Spectrometry (LC/MS) Analysis

*Escherichia coli* (ATCC25922), *S. aureus* (ATCC29213), and *P. aeruginosa* (ATCC9027) were incubated until OD(600) = 0.6 at 37°C, and then harvested by centrifugation at 6,000 rpm for 10 min at 4°C and re-suspended in PBS of 5 × 10^8^CFU/ml. The 1 ml of *E. coli* (ATCC25922) was added in 2-ml round-bottomed tubes; then, 0.5-μg EH was added. The mixture solution was incubated at 37°C for 1 h, and then centrifugated for 20 min at 6,000 rpm at 4°C. *P. aeruginosa* (ATCC9027) and *S. aureus* (ATCC29213) was processed in the same way. Finally, the supernatant was collected and 1:10 diluted with ultrapure water for subsequent test.

The supernatants were analyzed by LC/MS using Shimadzu triple quadrupole mass spectrometer with LC-30AD System (LCMS-8050). The chromatographic separation was achieved using an analytical column: Eclipse Plus C18 (2.1 × 150 mm, Agilent). The method for MRM was established on Labsolutions LCMS Ver 5.6. The mass spectrometer heating gas and drying gas flows were both at 10 L/min, with a nebulizing gas flow at 2 L/min. The heat block temperature was 400°C, and the interface temperature was 300°C. All compounds were analyzed using ESI in positive ionization mode, and two transitions in multiple reaction monitoring (MRM) mode were acquired for each analyte. The EH can produce two fragment ions with m/z of 231.10 and 204.10, corresponding to the CE value of −40 v, and −50 v. The standard curve solutions of EH at concentration of 0.001, 0.00625, 0.0125, 0.0025, 0.005, and 0.01 mg/L were prepared with the ultrapure water.

### Animal Experiments

To evaluate the therapeutic effects of these compounds on animals, female ICR mice (7 weeks) were obtained from SPF (Beijing) Biotechnology Co., Ltd. The study was carried out in strict accordance with the Regulations for the Administration of Affairs Concerning Experimental Animals of Hubei Province and the Regulations for Administration of Affairs Concerning Experimental Animals of China. All the experiment protocols and operation techniques were approved by the Committee for Protection, Supervision, and Control of Animal Experiments, Huazhong Agriculture University (HZAUMO-2019-011).

Animal experiments were carried out as described in a previous study, with some modifications (Ma L. et al., 2016). *E. coli* (42) was incubated until OD(600) = 0.6 at 37°C, and then harvested by centrifugation at 6,000 rpm for 10 min at 4°C, and then re-suspended in PBS. EH was dissolved into 5 mg/ml mother liquor with DMSO, and mother liquor was diluted to 0.5 mg/ml with 20% DMSO before injection. Mice (10 per group) were intraperitoneally injected with 200 μL of bacterial suspension (1.25 × 10^9^ cells/mL). At 1 h post-infection with *E. coli* (42), mice were intraperitoneally injected with EH at a dose of 5 mg/kg/day for 3 days. The untreated group (ten mice per group) received 0.2 mL 20% DMSO. After treatments, the mortality rate of the mice was observed for seven consecutive days. To investigate bacteria load and anti-inflammatory effect, the mice (five per group) were intraperitoneally inoculated with the equivalent amount of bacterial suspension. The injection procedure was performed as described above. Control group was only injected twice of PBS. At 12 h post-infection with *E. coli*, bacteria in the lung, spleen, kidney, and liver were counted, after these infected tissues were ground, diluted, and plated onto LA agar plate medium. The cytokines were quantified by using a sensitive electrochemiluminescence-based platform (Quickplex, Meso-Scale Discovery^®^, MD) (Starhof et al., 2018). To detect pathological change caused by bacteria, the left lobe of the lung was fixed in 4% paraformaldehyde for pathological examination. All mice were euthanized by CO_2_ inhalation. Cytokines measurement was repeated for three times.

### *In vivo* Toxicity Experiment

To assess the toxicity of EH to liver, kidney, and other major organs, mice were randomly divided into PBS control group and treatment group (five mice per group). Mice in the treatment groups were intraperitoneally injected with EH at a dose of 5 mg/kg/day for 3 days. At 12 h after the last administration, blood samples were collected from the anesthetized animals’ periorbital plexus. Biochemical analyses were performed using an automated analyzer (chemray 800, China). Visual examination was performed to detect abnormal signs. Alanine transaminase (ALT), aspartate transaminase (AST), creatinine, and urea nitrogen levels were analyzed (Huang et al., 2012).

### Statistical Analysis

All experimental data (*n* ≥ 3) are expressed as the mean ± SD. GraphPad Prism 8.0.2 was used for statistical analysis using two-tailed unpaired *t*-test.

## Results

### Antibacterial Activity of EH

We examined antibacterial activity of EH with a panel of Gram-positive and Gram-negative pathogenic bacterial species and found that EH exhibited potent antibacterial activity against *E. coli*, *S. aureus*, *K. pneumoniae*, *S. typhi*, and *L. monocytogenes*, with MIC ranging from 0.5m to 4 mg/L (Table 1). EH lacked activity against a *P. aeruginosa* strain (MIC ≥ 128 mg/L).

### Decatenation Assay

**FIGURE 1:**
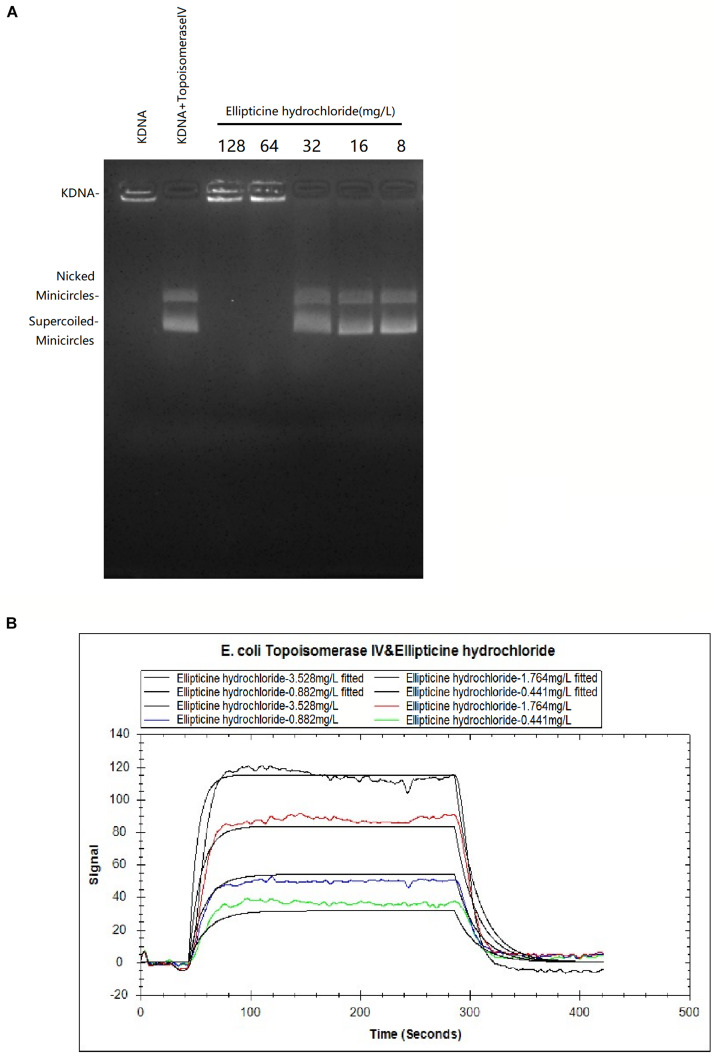
**(A)** The effects of EH (8 to 128 mg/L) on KDNA catalyzed by *E. coli* topoisomerase IV. Assays with intact kDNA but without *E. coli* topoisomerase IV, or kDNA treated with *E. coli* topoisomerase IV but without EH. The positions of intact kDNA at the origin, nicked kDNA minicircles, and supercoiled kDNA minicircles were indicated. **(B)** The binding kinetic analysis with LSPR between EH and *E. coli* topoisomerase IV. The equilibrium dissociation constant K_D_ was calculated as 7.64 × 10^–6^M with the Trace Drawer software (one-to-one model).

The inhibition of EH on topoisomerase IV activity was studied by decatenation assay (Figure 1A). Our results showed that 64 mg/L EH inhibited enzyme activity of *E. coli* topoisomerase IV, manifested by preventing kinetoplast DNA (kDNA) ring from breaking up.

### Localized Surface Plasmon Resonance

In LSPR assay, different concentration curves were obtained. The equilibrium dissociation constant K_D_ was calculated as 7.64 × 10^–6^M (Figure 1B) with the Trace Drawer software (one-to-one model), indicating that EH could effectively interact with the *E. coli* topoisomerase IV (a lower KD value indicated a higher affinity of the EH binding with *E. coli* topoisomerase IV) (Yu et al., 2018).

### Bactericidal Activity of EH Against *E. coli*

**FIGURE 2:**
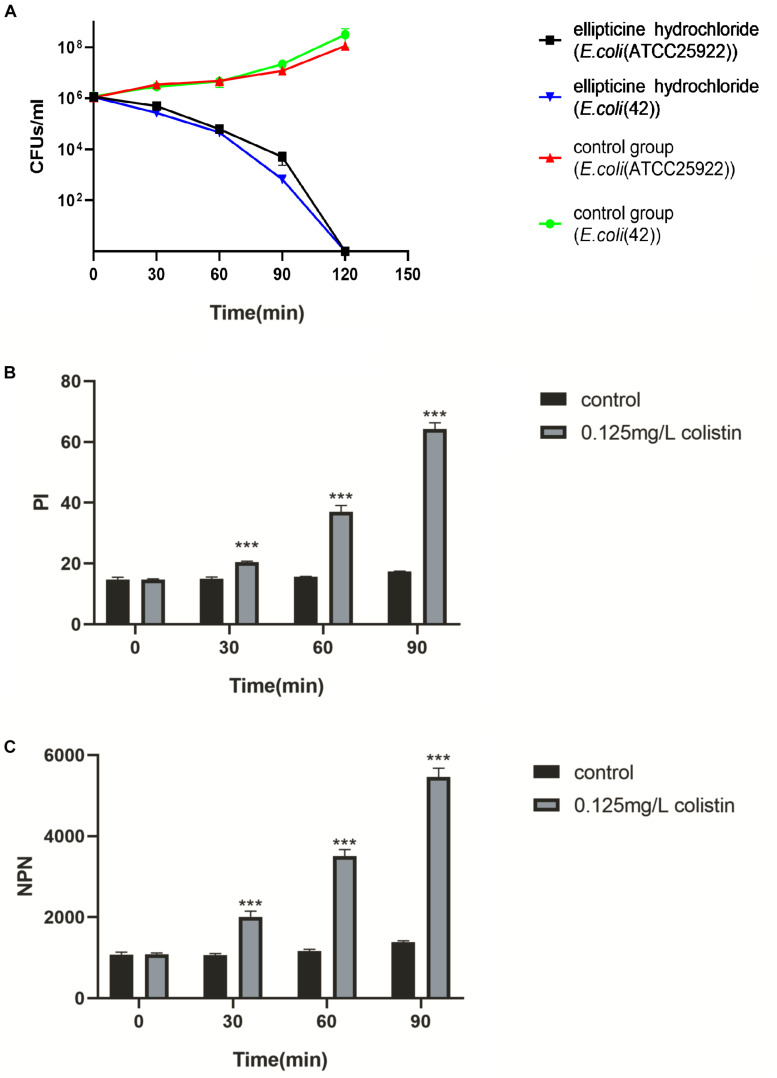
**(A)** The survival of *E. coli* (ATCC25922) and *E. coli* (42) in a broth culture treated with PBS, EH at a concentration of 4× MIC. **(B,C)** Inner and outer membrane permeabilization of colistin was measured by detecting the fluorescence intensity of PI **(B)** and NPN **(C)** in *E. coli* (ATCC25922). Statistical analysis was carried out by two-tailed unpaired *t*-test. *P < 0.05; **P < 0.01; ***P < 0.001.

To further examine the antibacterial nature of EH against *E. coli*, a time-kill assay was conducted at a concentration of 4× MIC. When MBC was no more than 4× MIC, an antibacterial compound is generally considered as bactericidal (French, 2006). EH was found to kill *E. coli* (ATCC25922) completely within 2-h exposure period (Figure 2A), indicating that EH was bactericidal against *E. coli* (Pang et al., 2019).

### Combination of EH With Colistin

The results of the combination showed that, compared with single administration of EH, the combination treatment decreased the MICs of EH in nine isolates (90%), and that the calculated FICIs displayed synergy effect in eight isolates (80%) and indifference effect in two isolates (20%), while no antagonism effect was detected (Table 2). Moreover, the combination of EH with ampicillin, tetracycline, cefotaxime sodium and levofloxacin showed no synergistic (Supplementary Table S1). *E. coli* outer membrane integrity was damaged by colistin in a time-dependent manner. The fluorescence intensity of PI (Figure 2B) and NPN (Figure 2C) was found to have increased at 1 h post-incubation, suggesting an increase in the membrane permeability of the bacteria. We speculated that colistin can destroy the integrity of the inner and outer membrane of bacteria which might be one of the important reasons for the synergy between the colistin and EH.

**TABLE 2:** Minimum inhibitory concentrations (MICs) and fractional inhibitory concentration indexes (FICIs) of EH and Colistin against 10 multidrug-resistant ExPEC isolates.

Strain no.	MIC (mg/L)			FICI	Relationship
	
	Colistin alone	Ellipticine hydrochloride combination	Colistin combination		
*E. coli* (1145)	8	0.25	0.5	0.3125	S
*E. coli* (13712)	8	0.25	0.5	0.3125	S
*E. coli* (1209)	8	0.25	0.5	0.3125	S
*E. coli* (1341)	16	1	8	1.5	I
*E. coli* (1704087)	8	0.5	1	1.125	I
*E. coli* (42)	8	0.5	1	0.375	S
*E. coli* (148121)	16	0.25	4	0.5	S
*E. coli* (1411060)	8	0.25	1	0.375	S
*E. coli* (1603043)	8	0.25	2	0.5	S
*E. coli* (1604040)	4	0.25	0.5	0.25	S

*S, synergy; I, indifference; A, antagonism.*

### Animal Experiments

**FIGURE 3:**
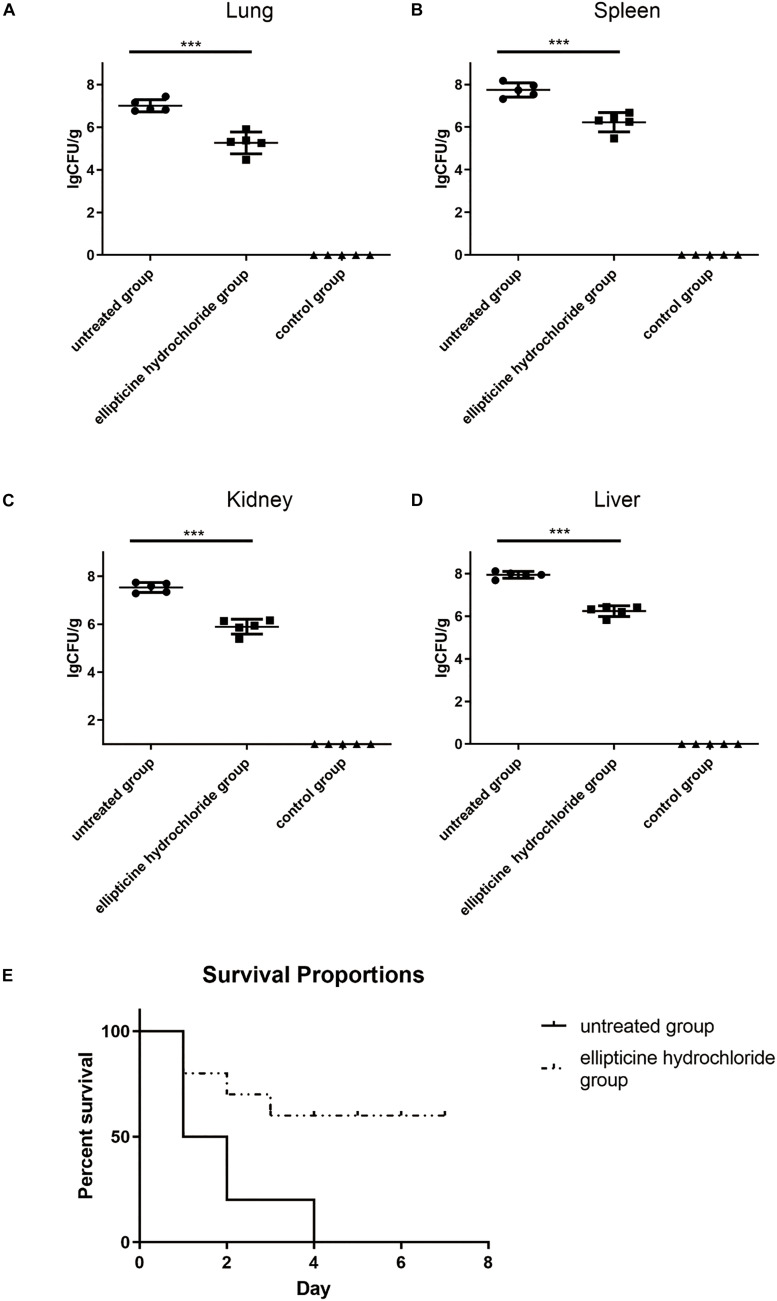
The bacterium number of *E. coli* (42) in the presence or absence of EH. Mice were intraperitoneally inoculated with 2.5 × 10^8^ CFU of *E. coli* (42). Bacterium number in the lung **(A)**, spleen **(B)**, kidney **(C)**, and liver **(D)** was counted at 12 h post-infection. Statistical analysis was carried out by two-tailed unpaired *t*-test. ***P < 0.001. **(E)** The roles EH played in the survival rate of *E. coli* (42)-infected mice.

*In vivo* antimicrobial activity was primarily detected according to the mice survival rate, bacterial titers in tissues, attenuation of lung injury, and the decrease in the pro-inflammatory factors. The results indicated that EH administration significantly increased the survival rate from 0 to 60%, compared to that of the untreated group (Figure 3E). Based on the bacteria counting, the increase in survival rate was related to the decreased bacterial titers in liver, lung, spleen, and kidney tissues from infected mice. On average, more than 1.5 log unit decrease in CFU was observed in the lung (Figure 3A), spleen (Figure 3B), kidney (Figure 3C), and liver (Figure 3D) tissues from the mice treated with EH.

**FIGURE 4:**
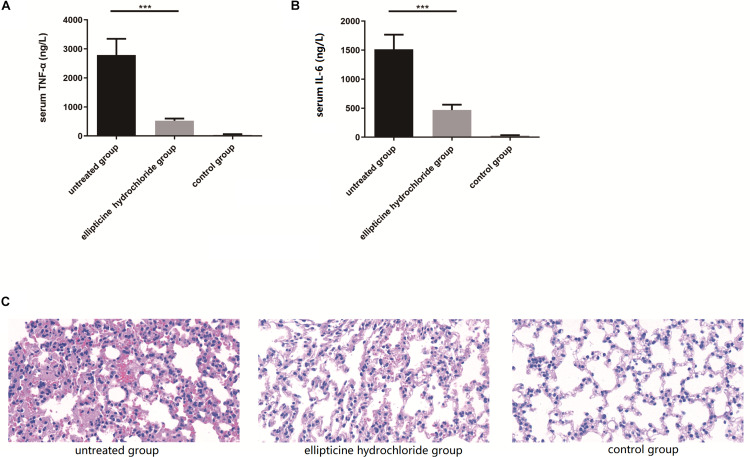
EH reduced the production of pro-inflammatory cytokines TNF-α **(A)** and IL-6 **(B)** induced by intraperitoneal injection of *E. coli* (42) in ICR mice. Statistical analysis was carried out by two-tailed unpaired *t*-test. ***P < 0.001. **(C)** At 12 h after infection, pathological examination of lung tissues of the infected mice.

As shown in Figure 4, the elevated levels of the IL-6 (Figure 4B) and TNF-α (Figure 4A) induced by infection were significantly decreased by more than 70% after the treatment with EH. Moreover, EH treatment alleviated other inflammatory manifestations that were observed in mice models with H&E staining, including inflammatory cell infiltration, dilatation of pulmonary vessels, alveolar interstitial congestion, and edema (Figure 4C).

Our results showed that EH exhibited good anti-inflammatory and antimicrobial activity in mice infected with colistin-resistant *E. coli*.

### *In vivo* Toxicity Evaluation

Antibacterial treatment is different from the treatment for cancers or chronic diseases since patients do not need long-term medications. Table 3 presented the comparison of the blood indexes of liver and kidney functions (urea nitrogen, creatinine, ALT, AST) between the treatment group (EH at the dose of 5 mg/kg/day) and the control group. As shown in the Table 3, the alterations in the functional parameter levels were not significant, which indicated no severe damage was induced by EH treatment. Additionally, no weigh loss or other obvious comorbidities were observed in the treated mice.

**TABLE 3:** Liver and kidney functions in the blood of mice in the control and treated groups.

Treatment^a^	ALT (U/L)^b^	AST (U/L)^b^	Creatinine (μmol/L)	Urea nitrogen (mmol/L)
Control	37.82 ± 1.40	94.91 ± 1.42	45.69 ± 0.867	12.32 ± 1.28
Ellipticine	37.21 ± 1.68	92.66 ± 1.78	46.02 ± 1.065	11.40 ± 1.15
hydrochloride	(*p* > 0.05)	(*p* > 0.05)	(*p* > 0.05)	(*p* > 0.05)

*^*a*^Mice (*n* = 5 in each group) treated with PBS or the EH (5 mg/kg) once daily for 2 days. ^*b*^ALT, alanine transaminase; AST, aspartate transaminase; U/L, international units per liter.*

### Compound Content Determination of Supernatant

**FIGURE 5:**
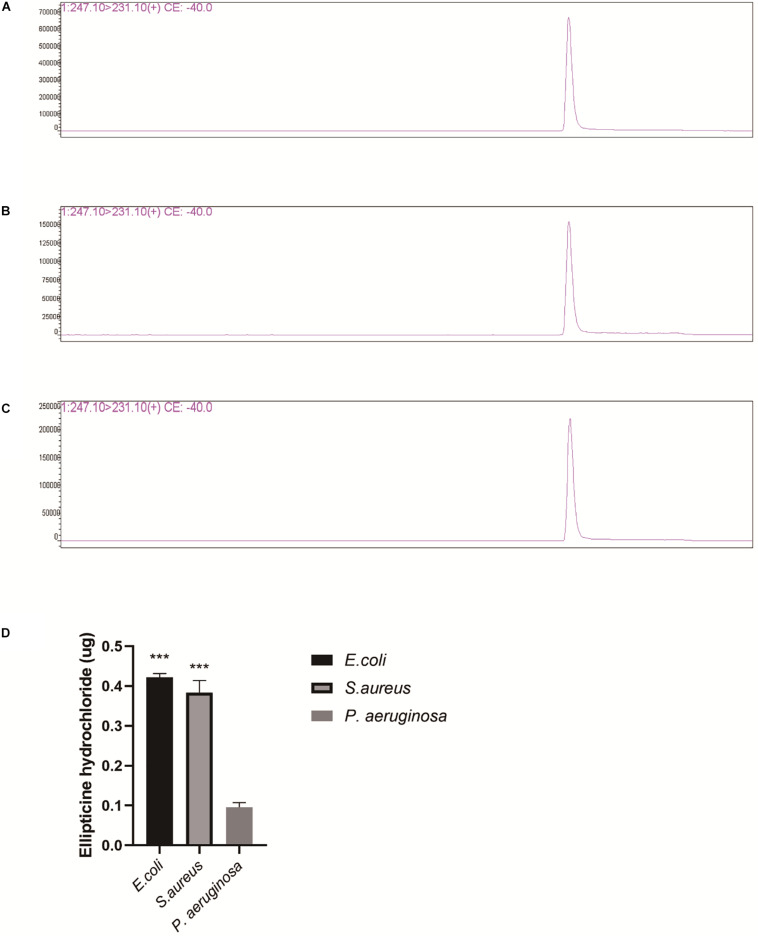
LC/MS analysis of EH (Repeat each sample three times and display a representative result.). **(A)** *P. aeruginosa* supernatant, **(B)** *E. coli* supernatant. **(C)** *S. aureus* supernatant. **(D)** EH concentration was calculated by standard curve. The measured amount was subtracted from original amount (0.5 μg). The figure presented is the amount of the EH entering the bacteria. Statistical analysis was carried out by two-tailed unpaired *t*-test. *P < 0.05; **P < 0.01; ***P < 0.001.

The inhibitory concentration of EH for the activity of topoisomerase IV *in vitro* was much higher than MIC of EH for most bacteria. Based on it, we speculated that the reason for this difference might lie in that the drug accumulation in bacteria caused drug concentration in bacteria to be much higher than that in the environment. This might also explain why EH was are ineffective against some bacteria with poor permeability, such as *P. aeruginosa*. Drug concentration of supernatant can be observed from the chromatograms (Figures 5A–C). Finally, the amount of supernatant of drugs was subtracted from the original amount of drugs, the result is the amount of drugs that go into the bacteria. As we guessed, the content of EH in *E. coli* and *S. aureus* was much higher than that in *P. aeruginosa* (Figure 5D). This is consistent with previous reports (Pang et al., 2019).

## Discussion

Traditionally, colistin resistance was thought to be associated with a chromosomal-related mechanism, and it could not horizontally transfer (Johansen et al., 2008; Moffatt et al., 2010; Arduino et al., 2012; Park et al., 2015). However, in 2016, the *mcr-1* gene was first found in *E. coli* isolates, and this finding has become a global concern. Subsequently, more seriously, the transferable colistin resistance mediated by *mcr-1* was reported in many countries (Cannatelli et al., 2016; Du et al., 2016; Elnahriry et al., 2016; Rapoport et al., 2016). The infection outbreaks of MDR *E. coli* in human or animal pose a huge threat to public health and safety. It has been estimated that there are at least 700,000 deceases every year worldwide due to infection with antibiotic-resistant bacteria, and this decease number will increase to 10 million every year by 2050 without new treatment strategies (O’Neill, 2014). Therefore, it is especially urgent to find new antibacterial drugs.

Inflammation is one of the serious consequences of bacterial infections. Lipopolysaccharide (LPS) (endotoxin) is the main component of the outer membrane of Gram-negative bacteria. LPS plays an important role in the pathophysiology processes of inflammation, sepsis, and shock caused by Gram-negative bacteria. During infection, LPS is released from the bacteria into the bloodstream, and it may cause severe and unnecessary irritation to the host’s immune system, leading to sepsis and septic shock in the patient (Ma L. et al., 2016). In many cases, the current therapy for infection involves the application of anti-inflammatory and antibacterial drugs (Mandragos et al., 2017). Previous studies reported that ellipticine exerted anti-inflammatory effects on the activated macrophages by inducting autophagy and inhibiting NF-κB signaling (Chen et al., 2019). In our study, EH was found to significantly increase survival rate by about 60% and reduce lung damage in *E. coli-*infected mice. These are consistent with the results of bacterial susceptibility test *in vitro*. EH showed potent bactericidal activity against extraintestinal pathogenic *E. coli*, which is resistant to various antimicrobials, including cephalosporins, tetracycline, penicillin, aminoglycosides, chloramphenicol, and colistin. EH has a good antibacterial broad-spectrum. The MIC for most of the tested bacteria was between 0.5 and 4mg/L. But decatenation assay showed that EH can inhibit *in vitro* the activity of purified *E. coli* topoisomerase IV at 64 mg/L, which is much higher than the inhibitory concentration for most bacteria. The report of Yague et al. (2002) also reflects this phenomenon. We speculated that the reason for this difference is that different bacteria have different concentrations of drugs, and the concentration of drugs in some bacteria may be much higher than that in the external environment. Subsequent LC/MS analysis confirmed our conjecture. The amount of EH entered into the bacteria (*E. coli* and *S. aureus*) is much higher than entering into the *P. aeruginosa*.

Drug combination is one of the important means to treat multidrug-resistant bacterial infection (Okdah et al., 2018). We found that co-administration of EH with colistin exhibited synergy effect in eight isolates (80%), with an indifferent effect in two isolates (20%). But the combined effects of ampicillin, tetracycline, cefotaxime sodium, and levofloxacin with EH *in vitro*, all test strains showed indifference (Supplementary Table S1). According to the experiment, we found that colistin-induced can destroy outer and inner membrane permeability. One possible explanation for the synergy effect might be that colistin greatly increased the permeability of the bacterial cell membrane, thereby enhancing the penetration of EH.

The high toxicity and side effects of EH have prevented this group of drugs entering clinical practice until now. It was reported that the molecular mechanism of ellipticine involved DNA intercalation and interference with the activity of topoisomerase II with different cytotoxic effects (Scheila et al., 2004). But inhibitory concentration of ellipticine for human topoisomerase II was more than 1,231.56 mg/L (Vann et al., 2016), far higher than that for bacterial topoisomerase IV. Moreover, it was also reported that healthy tissues of rats treated with ellipticine possessed effective DNA repair systems to remove certain lesions; based on this finding, it was further speculated that ellipticine might have a relatively low genotoxic side effects during cancer treatment in humans (Stiborová et al., 2014). Meanwhile, our study found that EH did not cause liver and kidney dysfunction at the dose-level of effective treatment in infected mice. EH is a promising candidate that deserves further research as a treatment option for multidrug-resistant *E. coli* infection.

## Data Availability Statement

All datasets generated for this study are included in the article/Supplementary Material.

## Ethics Statement

The study was carried out in strict accordance with the Regulation for the Administration of Affairs Concerning Experimental Animals of Hubei Province and Regulations for the Administration of Affairs Concerning Experimental Animals of China. All the experiment protocols and operation techniques were approved by the Committee for Protection, Supervision and Control of Animal experiments, Huazhong Agriculture University (HZAUMO-2019-011).

## Author Contributions

HL and ML were the principal experimenters with assistance provided by CW, WL, GW, and WD. HL and ML conducted the data analyses. CT, HC, HL, and XW were involved in the study conceiving and designing. HL and CT wrote the manuscript.

## Conflict of Interest

The authors declare that the research was conducted in the absence of any commercial or financial relationships that could be construed as a potential conflict of interest.
