# Supplementary material for: Repurposing Ellipticine Hydrochloride to Combat Colistin-Resistant Extraintestinal Pathogenic E. coli (ExPEC)
Source: Front Microbiol. 2020 May 25;11:806. doi: 10.3389/fmicb.2020.00806 (PMC7262907; doi:10.3389/fmicb.2020.00806)
Supplement: Supplementary file 1 [file Data_Sheet_1.doc]

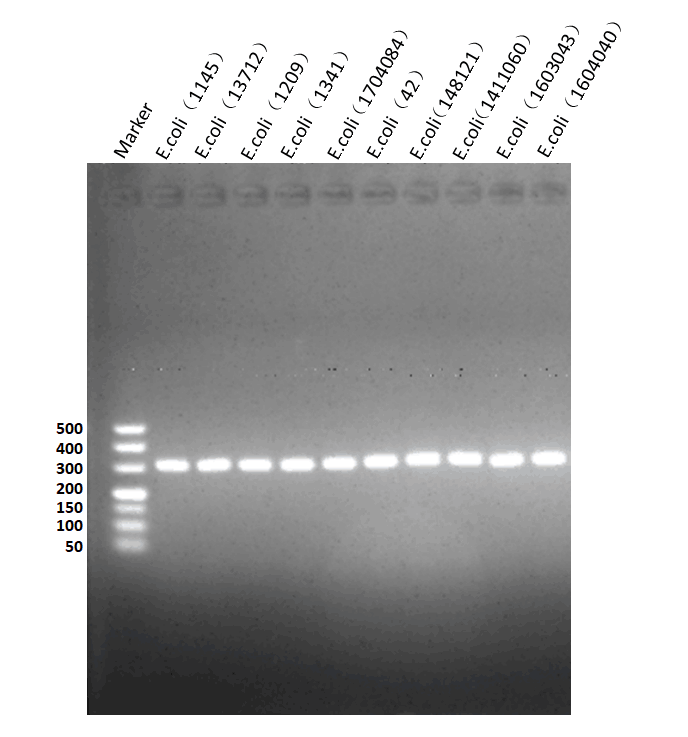


**Supplementary Figure S1.** **The evidence that these isolates contain the *mcr-1* gene.**

All *E. coli* strains were screened for the presence of the *mcr-1* gene by PCR using primers as previously described [1].

CLR5-F（5'-CGGTCAGTCCGTTTGTTC-3'）

CLR5-R（5'-CTTGGTCGGTCTGTA GGG-3'）

**Supplementary Table S1.** **Minimum inhibitory concentrations (MICs) and fractional inhibitory concentration indexes (FICIs) of ellipticine hydrochloride and four commonly used drugs against E.coli.**

| **Strain no.** | **MIC (mg/L)** | | |  |  |  |  |  |  |  | **FICI** | **Relationship** |
| --- | --- | --- | --- | --- | --- | --- | --- | --- | --- | --- | --- | --- |
|  | **AMP alone** | **TET alone** | **CTX alone** | **LEV alone** | **AMP combination** | **TET combination** | **CTX combination** | **LEV combination** | **EH alone** | **EH combination** |  |  |
| *E.coli(ATCC25922)* | 4 |  |  |  | 2 |  |  |  | 1 | 1 | 1.5 | I |
| *E.coli(ATCC25922)* |  | 1 |  |  |  | 1 |  |  | 1 | 0.25 | 1.25 | I |
| *E.coli(ATCC25922)* |  |  | 0.12 |  |  |  | 0.06 |  | 1 | 0.5 | 1 | I |
| *E.coli(ATCC25922)* |  |  |  | 0.06 |  |  |  | 0.015 | 1 | 1 | 1.25 | I |
| *E.coli*(42) | 16 |  |  |  | 16 |  |  |  | 2 | 0.5 | 1.25 | I |
| *E.coli*(42) |  | 64 |  |  |  | 32 |  |  | 2 | 1 | 1 | I |
| *E.coli*(42) |  |  | 2 |  |  |  | 1 |  | 2 | 1 | 1 | I |
| *E.coli*(42) |  |  |  | 16 |  |  |  | 8 | 2 | 1 | 1 | I |
| *E.coli*(13712) | 32 |  |  |  |  | 8 |  |  | 1 | 1 | 1.25 | I |
| *E.coli*(13712) |  | 32 |  |  |  |  | 32 |  | 1 | 0.5 | 1.5 | I |
| *E.coli*(13712) |  |  | 4 |  |  |  |  | 4 | 1 | 1 | 2 | I |
| *E.coli*(13712) |  |  |  | 2 |  |  |  | 2 | 1 | 0.5 | 1.5 | I |
| *E.coli*(1604040) | 64 |  |  |  | 32 |  |  |  | 2 | 2 | 1.5 | I |
| *E.coli*(1604040) |  | 64 |  |  |  | 64 |  |  | 2 | 1 | 1.5 | I |
| *E.coli*(1604040) |  |  | 2 |  |  |  | 2 |  | 2 | 1 | 1.5 | I |
| *E.coli*(1604040) |  |  |  | 4 |  |  |  | 2 | 2 | 1 | 1 | I |
| *E.coli*(1603043) | 32 |  |  |  | 16 |  |  |  | 1 | 1 | 1.5 | I |
| *E.coli*(1603043) |  | 32 |  |  |  | 32 |  |  | 1 | 1 | 2 | I |
| *E.coli*(1603043) |  |  | 2 |  |  |  | 2 |  | 1 | 0.5 | 1.5 | I |
| *E.coli*(1603043) |  |  |  | 4 |  |  |  | 2 | 1 | 1 | 1.5 | I |

Abbreviations: AMP, ampicillin; CTX, cefotaxime sodium; TET, tetracycline; EH, ellipticine hydrochloride; LEV, levofloxacin.

# Liu YY, Wang Y, Walsh TR, Yi LX, Zhang R, Spencer J, et al. Emergence of plasmid-mediated colistin resistance mechanism *MCR-1* in animals and human beings in China: a microbiological and molecular biological study. Lancet Infect Dis (2016) Feb;16(2):161-8.4.

.
